# Reply: Capecitabine and mitomycin C in patients with metastatic colorectal cancer resistant to fluorouracil and irinotecan

**DOI:** 10.1038/sj.bjc.6603022

**Published:** 2006-02-21

**Authors:** G Chong, D Cunningham

**Affiliations:** 1 Royal Marsden Hospital, G1 Cancer/Lymphoma Unit, Downs Road, London and Surrey, Sutton, SM2 5PT, UK

**Sir**,

We acknowledge Dr Alliot's letter and would like to respond to the many points raised. Firstly, we would like to reiterate our comments in the discussion that this regimen represents a viable option for patients with metastatic colorectal cancer who have previously received fluorouracil and irinotecan (Chong *et al*, 2005). The results of our study should not necessarily be extrapolated to patients who have previously received oxaliplatin or targeted agents.

For a single-arm study of third-line therapy for advanced colorectal cancer, the utility of biochemical prognostic factors in interpreting treatment efficacy is likely to be small. Therefore, these data have not been included. Of the 36 patients enrolled, 31 had liver metastases, and in eight patients, the primary tumour was *in situ*. Nevertheless, the treatment efficacy of a third-line regimen is not likely to be influenced by whether patients initially presented with synchronous or metachronous metastatic disease. With regard to the first-line 5-fluorouracil (5FU) regimen, a majority of patients received bolus 5FU/leucovorin (LV) according to the Mayo schedule; two patients received LV5FU2. There is no evidence that LV5FU2 *vs* bolus 5FU/LV influences the efficacy of subsequent chemotherapy.

The limited efficacy of capecitabine monotherapy for patients who are 5FU-refractory has been well demonstrated previously (Hoff *et al*, 2004). However, there are preclinical data suggesting that the addition of mitomycin C (MMC) to capecitabine may be synergistic. In patients with untreated advanced colorectal cancer, the addition of MMC to capecitabine has been shown to produce higher response rate than expected with capecitabine alone, (Rao *et al*, 2004). The 15.4% response rate for the combination of MMC and capecitabine observed in our study of pretreated patients is therefore consistent with these data.

There are substantial data on the incidence of haemolytic uraemic syndrome (HUS) in patients treated with MMC. The incidence for patients receiving less than 30 mg m^−2^ is very low (Ross *et al*, 1997, 2002; Tebbutt *et al*, 2002). However, we agree that patients receiving MMC, regardless of cumulative dose, should be closely monitored for the development of HUS.

Clearly, our data are not directly applicable to patients who have received first- or second-line oxaliplatin, bevacizumab or cetuximab. However, for patients who have not had (and will not have) access to these drugs, the combination of capecitabine and MMC is a potentially active one for patients with advanced colorectal cancer.
